# ACL Injury Etiology in Its Context: A Systems Thinking, Group Model Building Approach

**DOI:** 10.3390/jcm13164928

**Published:** 2024-08-21

**Authors:** Charis Tsarbou, Nikolaos I. Liveris, Sofia A. Xergia, George Papageorgiou, Joanna Kvist, Elias Tsepis

**Affiliations:** 1 Physiotherapy Department, School of Health Rehabilitation Sciences, University of Patras, 26504 Patras, Greece; ctsarmpou@upatras.gr (C.T.); n.liveris@upatras.gr (N.I.L.); tsepis@upatras.gr (E.T.); 2 SYSTEMA Research Centre, European University Cyprus, 2404 Nicosia, Cyprus; g.papageorgiou@euc.ac.cy; 3 Unit of Physiotherapy, Department of Health, Medicine and Caring Sciences, Linköping University, 58183 Linköping, Sweden; joanna.kvist@liu.se

**Keywords:** knee injury, ACL injury, risk factors, prevention strategies, systems thinking, system dynamics

## Abstract

**Background/Objectives**: Given the complex nature of Anterior Cruciate Ligament (ACL) injury, it is important to analyze its etiology with suitable approaches in order to formulate intervention strategies for effective prevention. The present study employs system thinking techniques to develop a Causal Loop Diagram (CLD) Model for investigating the risk factors for ACL Injury (CLD-ACLI), through a Group Model Building approach. **Methods**: A two-stage procedure was applied involving a comprehensive literature review followed by several systems thinking group-modeling co-creation workshops with stakeholders. **Results**: Based on input from experts and stakeholders, combined with the latest scientific findings, the derived CLD-ACLI model revealed a series of interesting complex nonlinear interrelationships causal loops between the likelihood of ACL injury and the number of risk factors. Particularly, the interaction among institutional, psychological, neurocognitive, neuromuscular, malalignment factors, and trauma history seem to affect neuromuscular control, which subsequently may alter the biomechanics of landing, predisposing the ACL to injury. Further, according to the proposed CLD-ACLI model, the risk for injury may increase further if specific environmental and anatomical factors affect the shear forces imposed on the ACL. **Conclusions**: The proposed CLD-ACLI model constitutes a rigorous useful conceptual presentation agreed upon among experts on the dynamic interactions among potential intrinsic and extrinsic risk factors for ACL injury. The presented causal loop model constitutes a vital step for developing a validated quantitative system dynamics simulation model for evaluating ACL injury-prevention strategies prior to implementation.

## 1. Introduction

ACL injury in team-ball sports is one of the most serious sports injuries, provoking several consequences at the personal, institutional, and societal levels. These include the long-term effects on the athletes’ health, athletes and teams’ performance, as well as the considerable financial burden on the team and society, constituting strong incentives for investment in effective injury-prevention policies [1,2]. More than half of all ACL injuries in team-ball sports are noncontact [3]; therefore, they are considered potentially preventable. Although various injury prevention programs have been established and proven to be effective in decreasing ACL injury rates [4], trends in ACL injury rates in the last few decades have been on the rise [5,6]. In this regard, the reasons for effective prevention strategies are being discussed, and special attention has been paid to the methodologies used to understand the etiology of ACL injuries [7].

To facilitate the understanding of the etiology of ACL injury and suggest solutions for its prevention, researchers have been able to categorize potential risk factors into two main areas. Internal risk factors involve athletic capacities [8,9] and external risk factors include all those interactions between the athlete and the environment [8,10]. These factors can be further divided into those that can be modified through specific interventions, named modifiable factors, and those that cannot change (non-modifiable) [8]. Intrinsic and modifiable factors have attracted the attention of clinicians and researchers worldwide, and many exercise injury prevention programs have been established that focus on modifying the neuromuscular and biomechanical risk factors [4,11]. However, it is still up to investigate the level of influence of these well-studied intrinsic and modifiable factors on the risk of ACL injury.

A recent meta-analysis by Collings et al. [12] found 19 risk factors for ACL injury in female team sports players, suggesting that only three are influential. These factors included previous ACL injury (OR = 3.94, 95% CI 2.7–7.50), bipedal increased postural sway (locus length) during a 30 s stance with eyes open (SMD = 0.58, 95% CI 0.02–1.15), and increased body mass (SMD = 0.25, 95% CI 0.12–0.39) [12]. However, it appears that factors do not behave as significant when studied in isolation and may prove of significant impact on injury occurrence when they are examined in combination with each other [13,14]. For example, a coexisting excessive lateral trunk excursion during landing along with knee valgus during a single leg drop vertical jump was a significant predictor of knee injury in female athletes [13]. Furthermore, athletes with a combination of decreased notch width, increased anteroposterior laxity, and increased body mass index presented a relative risk 21.3 times compared with controls, while these variables, when studied in isolation, had a relative risk of 3.8, 2.6, and 2.0, respectively. Likewise, the coexistence of decreased medial tibial depth, increased lateral tibial plateau slope, and increased medial tibial slope resulted in an odds ratio of 4.18, which is considerably higher than when it is considered in isolation [14]. Multivariable risk factor analysis enhanced the traditional process of investigating the effect of risk factors in musculoskeletal injuries based on linear statistical models and improved the understanding of the etiology of the injury [15,16].

Recent evidence suggests analysis of sports injuries as a complex phenomenon and proposes ecological and dynamic system approaches to better understand the etiology of injuries [7,17,18]. Comparisons of the statistical methods used (linear or non-linear) on the risk factors for secondary ACL injury indicated that non-linear methods identified more risk factors than the traditional linear approach [19,20]. Non-linear methods are supported by the fact that the factors contributing to sports injury interplay dynamically increase or decrease the risk of injury across seasons [17]. For this reason, the importance of initially conducting qualitative research is emphasized as it can provide deeper insight into the injury problem [21,22,23]. Some qualitative research can fill the gaps in the understanding process, providing a more naturalist approach than quantitative methods, trying to find a unique assumption that can be generalized [21]. 

Given the complex nature of sports injuries, it is important to be able to investigate their etiology through approaches that enable us to do so [24]. Systems Thinking and the System Dynamics (SD) methodology [25] can complement the current reductionist approach with a more holistic realistic analysis, enabling the depiction of the non-linear relationships among the risk factors and any emerging patterns leading to an injury [24]. Specifically, the system dynamics methodology includes specific steps [26] that should be implemented to model the risk and evaluate the propensity for injury. The methodology for formulating an SD model requires qualitative and quantitative procedures. The qualitative steps include an initial group discussion among stakeholders on the factors affecting the risk for ACL injury and developing a dynamic hypothesis of factors’ interaction [25]. The quantitative steps include examining factors’ interactions using real data, formulating a quantitative SD simulation model, testing the model, and examining different scenarios [25]. The first key step during the qualitative process involves the construction of a Casual Loop Diagram (CLD) model using a group modeling method to describe the positive and negative feedback loops that sustain the behavior of the ACL injury complex system [24,25]. Therefore, the objective of the present study was to qualitatively model and visually describe the risk of ACL injury, by the composition of an ACL injury CLD.

## 2. Materials and Methods

A two-stage methodological procedure was followed to develop the ACL injury Casual Loop Diagram (ACLI-CLD) model. The first stage involved a review of the latest scientific articles in the field aiming at gathering an initial set of risk factors contributing to ACL-related injuries. The second stage involved developing the actual causal loop model via Systems Thinking techniques, which incorporated input from the first stage, as well as input from experts and stakeholders in the field of ACL injuries, via a rigorous Group Model Building approach. This is viewed as a fundamental step of our research project aimed at formulating a quantitative SD model for the risk factors of ACL injuries. The second stage also incorporated the experiences from some previous work performed considering all acute non-contact lower limb injuries and hamstring injuries [26,27].

In the first stage, a comprehensive systematic literature review was conducted, including recent systematic reviews, meta-analyses, and studies that explored the risk factors contributing to ACL injuries in team sports. In this regard, two databases (SCOPUS and PubMed) were searched for studies that retrospectively and prospectively examined the factors linked to ACL injuries. To identify the most important studies specific relevant keywords were used. These included: “Anterior cruciate ligament”, “injury or rupture or tear “risk factors or etiology”. Since we found that the literature reviews of the published recent systematic reviews conducted until 2016, we executed another search to identify possible further risk factors from 2017 until May 2024. This literature review and search strategy was not systematic, as its purpose was to integrate the pivotal risk factors for ACL injury to be discussed in GMB workshops. After conducting an extensive literature review and formulating the initial CLD for ACL injuries by the principal investigator, and the participants were invited to discuss the model and express their opinions regarding the nonlinear interrelationships presented in the initial CLD. 

In the second stage, the Group Model Building (GMB) methodology [28,29] was applied, where several structured workshops with stakeholders were conducted to promote collaborative and insightful examination of ACL injury etiology [23,28]. The GMB process for the formulation of ACLI-CLD is presented in Table 1. During this phase, experts and stakeholders across the spectrum of sports injury prevention were invited to participate actively in the formulation of the ACLI-CLD. If necessary and more information was needed further literature reviews were conducted to enhance the elements of the model.

### 2.1. Participants

Twelve participants with various expertise (sports psychologists, strength and conditioning coaches, physiotherapists, and doctors) were invited to participate in the construction of the ACLI-CLD. Due to scheduling limitations, four stakeholders (one sports psychologist, two conditioning trainers, and one sports physical therapist) were excluded from participating in this study. As a result, four stakeholders participated in the GMB workshops. The absence of sports psychologists and conditioning trainers from the GMB workshops hinders the ACLI-CLD since their insights were not incorporated into the model. Nonetheless, the small groups of participants in the GMB workshops foster communication among the modeling process members, enabling a dynamic and in-depth discussion [23]. Four members constituted the core modeling team and four members of the stakeholder team. The core modeling team included two clinical physiotherapists pursuing Ph.D. studies in sports injury prevention, one academic physiotherapist with expertise in sports injury prevention and rehabilitation with extensive research and clinical experience, as evidenced by relevant published literature, and one academic expert in System Dynamics modeling. The stakeholder team consisted of two academic physical therapists with extensive research and clinical work in sports injury prevention; one orthopedic surgeon with extensive clinical and research experience in sports injuries who serves as the head of medical staff for a Greek first-division football club, and one sports physiotherapist experienced in sports injury rehabilitation who serves in the medical team of a Greek first-division football club. The primary consideration for incorporating stakeholders in the GMB workshops was that they were professionals who dealt with various facets of athlete health.

### 2.2. Procedures

Initially, three meetings of the core modeling team were conducted. At the first meeting, the risk factors that emerged from the literature review were presented and their possible interrelationships were discussed [29]. In the consecutive two meetings, the initially formulated CLD was discussed and edited [28,29] and the next meeting was organized with the integration of the stakeholders into the process. 

The core modeling team prepared a video presentation on the ACLI-CLD to introduce the scope of the meeting to invited participants. At this meeting, all the participants were involved in a detailed web discussion of each component of the ACLI-CLD, which lasted two hours. Each member of the core modeling team played a specific role: (1) the facilitators (SAX and GP), who guided the team and facilitated the procedure; (2) the modeler (CT), who edited the model; and (3) the recorder (NIL), who recorded the main conclusions that emerged through the process [28,29]. The main objective of the core modeling team was to engage stakeholders in conversations about the ACLI-CLD and incorporate their valuable insights into the model. The input provided by the stakeholders was essential in addressing the ACLI issue. The core modeling team carefully gathered and evaluated the feedback from the stakeholders to enhance the ACLI-CLD. After gathering feedback from the stakeholders, the core modeling team had a final meeting to review and modify the ACLI-CLD.

After a brief description of the objective of the research, the participants provided their consent to participate in the procedure. This study was approved by the Ethical Committee of the University of Patras and registered at ClinicalTrials.gov (NCT05430581). The Vensim software (PLE version 10.1.3, Ventana Systems, Harvard, MA, USA) was used to formulate the ACLI-CLD.

## 3. Results

**Figure 1:**
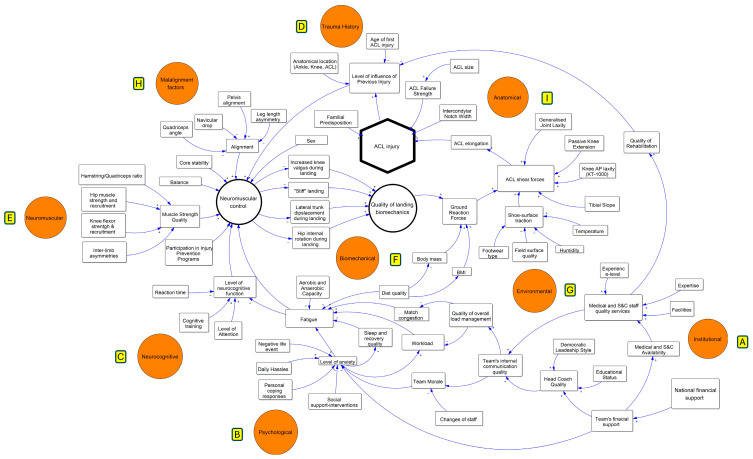
Conceptual ACL Injury Causal Loop Diagram explaining the nonlinear association of injury etiological factors. Risk factors were grouped into specific corresponding categories (named in the orange circus). In addition, the diagram shows whether the relationship between the two variables is positive or negative. A positive causal link (+) is created when an increase in a variable causes an increase to the other variable and a negative causal link (−) appears in the case of opposite polarities.

The literature review and subsequent sessions with stakeholders resulted in the creation of a conceptual ACLI-CLD model including several factors that contribute to ACL injury. As presented in the diagram of Figure 1, the ACL injury risk factors were grouped into specific corresponding categories (named in the red circus). In addition, the diagram shows whether the relationship between the two variables is positive or negative. A positive causal link (+) is created when an increase in one variable causes an increase in the other variable and a negative causal link (−) appears in the case of opposite polarities. 

### 3.1. General Characteristics of the ACLI-CLD Model

*Ιnstitutional*, *psychological*, *neurocognitive*, *malalignment* factors, and *trauma history* affect neuromuscular control, which may alter the biomechanics of landing and cutting, predisposing the ACL to injury (Figure 1). This risk can increase further if specific *environmental* (e.g., match congestion) and *anatomical* factors (e.g., joint laxity and tibial slope) affect the shear forces imposed on the ACL. *Institutional* factors considered the factors that concern the team’s function, such as the team’s internal communication quality, the coach’s leadership style, and the medical staff’s availability and quality (Figure 1 category A). *Psychological* factors include the level of anxiety, psychological fatigue, and the factors that affect this psychological condition of the athlete such as the negative life events and social support (Figure 1 category B). The *neurocognitive* factors include factors such as reaction time, cognitive training, and the level of attention which affect the level of neurocognitive function (Figure 1 category C). *Trauma history* factors include the history of previous injuries of the athlete (Figure 1 category D). *Neuromuscular* factors are those that are related to ACL protection such as hip and thigh muscle strength and activation, core stability, balance, and inter-limb symmetry (Figure 1 category E). *Biomechanical* factors include the factors that predispose the ACL to injury such as lateral trunk displacement and knee valgus during landing (Figure 1 category F). *Environmental* factors include the factors that increase the climatic conditions and surface types that affect the level of friction between the shoes of the athlete and the surface affecting the forces transmitted to the knee (Figure 1 category G). *Malalignment* factors concern the anatomical differences between the left and right sides of the athlete (Figure 1 category H). And finally, *anatomic* factors include characteristics of the knee joint and anatomy that affect the vulnerability of the ACL (Figure 1 category I). In the text below, the dynamic interactions of the factors are presented.

### 3.2. Factors’ Interactions of Landing Mechanism

The level of neuromuscular control of the lower limbs and trunk is reflected by the quality of landing biomechanics [30,31]. It is documented that ACL injuries occur during landing, pivoting, and cutting tasks [32,33] and its mechanism includes a combination of knee valgus; upright trunk posture along with low hip, knee, and ankle flexion angles; lateral trunk displacement; and large hip internal hip rotation and/or hip abduction during the initial contact of the foot [33]. Thus, abnormal biomechanical patterns during dynamic movements increase the shear forces of the ACL and thus the risk of ACL injury [34,35,36]. Some studies [33,34], incorporating neuromuscular training programs confirm this relationship [30,31,37,38,39]. The literature review and subsequent workshops suggest that several factors contribute to neuromuscular control. These include the level of physical and psychological fatigue, the level of previous injury and neurocognitive function, core stability, muscle strength quality, and all the factors included in the categories of malalignment and institutional factors. The characteristics of the neuromuscular control that affect the quality of landing biomechanics are presented in the following paragraphs, including *neuromuscular* factors and the indirect effects of *institutional*, *psychological*, and *malalignment* factors along with *trauma history* on ACL propensity of injury.

#### 3.2.1. Effect of Neuromuscular Control on Quality of Landing Biomechanics

A plethora of studies have confirmed the interrelationship of muscle strength with lower-limb biomechanics and ACL strain [31,37,40,41,42,43,44,45]. Specifically, the greater the hip muscle strength deficiency in all planes, the greater the dynamic knee valgus during single-leg landing [41]. Furthermore, evidence suggests that athletes with lower core stability, as examined by the side plank endurance test, present lower hip and knee flexion during jump landings [37]. Thus, poor neuromuscular control of the hip joint and trunk may increase the forces on the knee joint during landing and cutting tasks [31,42,43]. In addition, lower strength and activation of knee flexors decrease the posterior shear forces, limiting their protective function to the ACL during dynamic tasks [40,46]. Balanced quadriceps to hamstring muscle isokinetic strength is associated with lower knee valgus and lower ground reaction forces during landing [46]. The inability of the hamstrings to counteract the excessive shear forces of the quadriceps muscles and ground reactions during dynamic tasks may compromise ACL integrity [46,47]. Furthermore, a low quadriceps-to-hamstring muscle strength ratio and symmetry in both strength and ratio have been proven to increase the risk of ACL injuries [48,49].

#### 3.2.2. Effect of Neurocognitive, Institutional and Psychological Factors on Neuromuscular Control

The imbalance between stress and recovery can affect several aspects of the physical and psychological health of athletes and may increase their risk of injury [50]. The fatigue of the athlete affects neuromuscular control and the vulnerability to ACL injury negatively through its effect on neuromuscular control. Muscle fatigue has been suggested to alter landing biomechanics and load on the ACL, predisposing the lower limb to ACL injuries [35,51,52,53,54,55]. As physical fatigue increases, the internal ability to absorb loads during dynamic tasks declines, predisposing the ACL to injury [55]. Fitness level, including aerobic and anaerobic capacity, the quality of sleep, recovery, and diet [56] are factors that improve fatigue resistance. 

On the other hand, increased psychological stress can lead to a decline in cognitive functions, including the level of attention and reaction time, making the athlete unable to respond efficiently to somatosensory information and biomechanical demands when experiencing rapid changes in environmental conditions [57,58]. Practicing neurocognitive training when fatigued may prepare the neuromuscular system of the athlete to cope better with fatigue [50]. However, as athletes function as part of their team, the demands of their team could affect the level of fatigue; The stakeholders’ decision-making process regarding the training load and match congestion of each athlete contributes to the level of fatigue. Excessive and rapid increases in training loads increase the level of fatigue [59], resulting in biomechanical adaptations that predispose athletes to ACL injury [55].

In addition, pressuring behaviors from coaches, family, competitors, and even themselves subject athletes to continuous psychological stress in an attempt to satisfy their expectations [60]. This, in combination with the history of stressors (a breakup of a relationship, the death of a family member, etc.) along with insufficient social support, creates a web of relationships that affects the level of anxiety and, by extension, the neurocognitive function of the athlete [57]. The higher the team demands on the athlete, the higher the level of anxiety, which in turn contributes to an increase in psychological fatigue [61]. Each coach has a philosophy, educational background, and leadership style [61,62] that determine its quality. In teams that dominate values such as discipline, struggling for perfection and winning at all costs may lead to decisions where athletes’ health is compromised [61]. For this reason, special attention is given to the quality of internal communication among all key members of the team, especially medical staff and head coaches [62]. Stakeholders suggest that the higher the quality of communication, the higher the quality of the overall load management of the athletes and team morale. Stakeholders proposed the introduction of the parameter team morale which increases as the quality of communication improves. A higher team morale can alleviate the emotions of anxiety in athletes. However, when the cohesion of the team is compromised due to changes in team staff then the morale of the team falls. Finally, during the final workshops, special mention was made regarding the role of local and national policies on the safety of sports. Highlighting the financial support provided to teams for their proper functioning. 

#### 3.2.3. Effect of Malalignment on Neuromuscular Control 

*Malalignment* factors (Figure 1 category H), including leg length asymmetry, pelvic alignment, navicular drop, and quadriceps angle, have been shown to affect neuromuscular control. Increased navicular drop when combined with decreased quadriceps angle has been linked to delayed activation of the protective lateral hamstrings when subjected to external rotation perturbation while standing in a single-leg stance [63]. Furthermore, increased navicular drop and decreased quadriceps angle have been associated with ACL injuries [16]. Further leg length asymmetry and pelvic misalignment can cause an unequal distribution of weight, compromising the passive tissues of the knee during dynamic tasks [64].

#### 3.2.4. Effect of Trauma History Characteristics, Sex, and Participation in Injury Prevention Programs on Neuromuscular Control

Trauma history (Figure 1 category D) seems to be a strong determinant of subsequent ACL injury by modifying the complex interactions between other risk factors, as stated in the literature [12,65] and stakeholders’ opinions. The anatomical location of the injury (ankle and ACL) and the quality of rehabilitation determine the level of influence of previous injuries [12,66]. In addition, the lower the age (<25 years) of the first ACL, the greater the increase in the level of influence of previous injury [67]. Young athletes with a history of previous ACL injury have a 30–40 times greater risk of ACL injury than uninjured adolescents [67]. Insufficient restoration of functional limitations after surgery or injury that may increase the probability of a subsequent ACL injury may be limited if athletes have access to high-quality rehabilitation of the injury. The higher the quality of rehabilitation, the lower the level of influence of previous injuries on neuromuscular control. The quality of rehabilitation depends on the quality of the medical staff, which is a function of the availability of the medical team, the experience, and the expertise of its members. Family history is another factor in trauma history. Evidence suggests that a family history of ACL injury increases 2-times the risk of sustaining a primary or subsequent ACL injury in both males and females [68]. 

Participation in injury prevention programs has been proven to have a significant effect in reducing ACL injuries. The protective effect of these programs has been estimated to lead to a 50–53% reduction in ACL injuries [11,69]. Efficacious injury prevention programs incorporate strengthening, plyometric, and agility exercises along with education for proper landing technique [11]. Apart from the inclusion of exercise from at least these three categories combined with proper landing technique education, the injury prevention programs should be implemented 2 to 3 times per week during pre-season and in-season training [11].

Female athletes have a higher risk of ACL injuries than their male teammates [3]. Researchers have attempted to explain this disparity from a biological perspective, including anatomical, hormonal, and neuromuscular differences. In addition, cultural and social differences in the way girls grow and are dealt with in our society should not be ignored. The toys and activities of boys expose them more to stimuli facilitating neuromuscular adaptations, in contrast to girls. Furthermore, inequalities in support and access to medical services and facilities between female and male teams result in differences in physical status [70]. In female athletes, evidence suggests that anterior knee translation is higher during the ovulatory phase (days 10–14), followed by the luteal phase (days 15–28), compared with the follicular phase of the menstrual cycle (days 1 to 9). However, the periods during which most ACL injuries occur are not associated with hormonal increases in knee laxity. Most injuries occur during the first half of the menstrual cycle (pre-ovulatory phase) rather than during the luteal phase. Therefore, it is suggested that the effect of hormones on the ACL injury rate occurs through mechanisms other than knee laxity [71]. 

### 3.3. Effects of Anatomic and Environmental Factors on the Forces Transmitted to the ACL

Intrinsic non-modifiable *anatomic* factors affect the level of shear forces to which the ACL is subjected. As generalized joint laxity [16], knee joint laxity [72,73], and passive knee extension [16] are elevated, the ACL shear forces and risk of ACL injury are also elevated. Further anatomic characteristics, including the intercondylar notch width [74] and tibial slope [75], affect the level of shear forces [76,77]. Increased medial tibial plateau slope (MTPS) and lateral tibial plateau slope (LTPS) have been associated with an increased risk of ACL injury in males but not in females [75]. However, associations have been established between MTPS/LTPS in females and movement biomechanics that predispose the ACL to injury [76,78,79,80,81]. In addition, the morphology of the femoral intercondylar notch has been associated with ACL injury [74,82,83]. A stenotic intercondylar notch may accommodate an ACL of smaller size and thinner volume that can be torn at lower loads [84,85]. Moreover, during knee flexion and rotation, the ACL impinges more easily on the inner wall of the lateral femoral condyle [84].

Environmental factors like the level of rotational traction between the playing surface and the player’s footwear during cutting and turning affect the level of force to which the ACL is subjected. A trapped foot on the ground increases the force on the ACL and the risk of injury [86]. In a 3-year prospective study, athletes using edge cleat design shoes had the highest peak of rotational traction and 3.4 times increased ACL injury rate than the three other cleat designs combined [87]. Temperature and humidity affect the risk of ACL injury by influencing shoe-surface interactions [88,89]. Higher climatic and surface temperatures and dry conditions seem to increase shoe-surface traction, thereby increasing the risk of ACL injury [88,89].

## 4. Discussion

The present study aimed to formulate a CLD to identify and understand risk factors for an ACL injury. The formulated CLD demonstrates the complexity of the ACL injury etiology system. Multiple factors that positively or negatively affected the system were identified. This suggests that optimal interventions for ACL injury prevention should not be considered in isolation, and it presupposes a long-term strategy that negotiates multiple aspects of the aforementioned factors of the system. Understanding the dynamic interaction of ACL injury risk factors and designing appropriate preventive methods could shape relevant and effective intervention strategies and national policies for the support of teams and athletes. Effective injury prevention strategies require a holistic approach and collaborative work from key team partners including team staff, medical staff, athletes, and administrators.

Injury prevention programs mainly focus on the modification of neuromuscular and biomechanical risk factors associated with ACL injuries. Although these programs have been proven effective in improving the quality of landing and reducing ACL injury, the trends in ACL injury in the last 20 years are far from reassuring that sports injury prevention is moving towards the right direction [5,6]. A possible explanation for these trends may be that not all teams adopt injury prevention programs in their training routines and submit their athletes for preseasonal screening. Limited time availability to include specific prevention exercises during the training routine, financial barriers, and discrepancies among team staff members regarding priorities and needs may explain the condition [90]. In addition, existing injury prevention programs have been established based on current research methodologies on risk factors, attempting to link a single factor directly with an injury [4]. The current literature and the findings of the present study support that ACL injury is a complex phenomenon, and the identification of a single factor that predicts ACL injury is far from reality. Further, it seems that non-linear methods, especially qualitative methods including narrative reviews and structured workshops with stakeholders which are incorporated in the first steps of system dynamics methodology [23], are more inclusive than the traditional linear methods that prevail in the literature. For most variables, traditional linear methods did not find an association with ACL injuries [91,92,93]. This rationale can lead to an understatement of some factors along with faulty generalized conclusions that may result in the exclusion of specific parameters from an injury prevention plan.

The ACLI-CLD is an adaptive system in which changes in its individual parts can positively or negatively alter the susceptibility to injury. The neuromuscular characteristics of athletes adapt to the workload imposed, leading to positive or negative adaptations from the injury prevention perspective. However, throughout the season, many changes can occur, altering the behavior of the system, such as changes in team staff, environmental conditions, psychological mood, and level of fatigue. For this reason, frequent monitoring of athletes and the team’s progress is suggested to identify potentially negative adaptations to the workload that may lead to an ACL injury [50,59]. Athletes’ screening should be field-based, feasible to conduct, cost-efficient to implement on a large scale, and able to provide the necessary information with validity and reliability [94,95]. Furthermore, the evaluation of the team’s progress on injury prevention should be reevaluated on a regular basis through meetings organized by team members focused on injury prevention [90].

In the existing literature, there is an overemphasis on intrinsic-modifiable and anatomical factors. This may lead to labeling of the human body and towards an individual’s responsibility. The present ACLI-CLD provides a holistic approach to the problem, highlighting the role of the environment, and including the family, team, and society. Stakeholders introduced some new parameters that affect the system. Team morale, overall load management, medical staff quality, head coach quality, and financial support from teams and states constitute the new parameters that according to stakeholders’ opinions through various paths affect the internal qualities of the athlete. These proposed indicators are in line with the recent literature reviews highlighting the determining role of the environment on risk for injury [7,17,24,61]. A supporting social environment oriented to assist the teams in injury prevention initiatives and provide access to medical services and facilities are some of the basic factors that affect the internal qualities of the athlete, including stress response, decision-making, and movement patterns. Through a literature review, it was observed that the research community lacks studies that consider factors framing the environment of the athlete. Effective injury prevention strategies targeting injury-prevention-friendly environments require a better understanding of the mechanisms by which the environment, including the team and society, interacts with athletes.

In the present ACLI-CLD, the interrelationships among the factors, along with polarity, are presented. The magnitude of this effect has not yet been studied. In a complex system, small changes in some variables can significantly affect the function of the system, whereas large changes in other variables will have little effect on the system [7]. The size of the effect of the variables on the probability of ACL injury was far from that of the aim of the present study. The resulting model constitutes the first part of the research, aiming at the quantification of the interrelationships among factors and the formulation of a dynamic simulation model through system dynamics methodology [24] that will provide information regarding the propensity of ACL injury.

The ACLI-CLD model has some limitations that are important to acknowledge. To begin with, expanding the stakeholder base to include various specialties, such as coaches, players, and psychologists, could enrich the model’s development. However, constraints related to the availability and commitments of these professionals limited their participation in the GMB process. Expanding stakeholder participation could lead to more accurate factor interrelationships. Furthermore, the qualitative nature of the model necessitates additional efforts to quantify factor interactions, emphasizing the need to move toward a more quantifiable analysis. The “state-of-the-art” methodology of Structural Equation Modeling (SEM) can be employed to transform a qualitative Causal Loop Diagram into a quantitative SD model. Real data from athletes’ screenings including for instance strength, flexibility, core stability tests, questionnaires, workload, and biomechanical data can be analyzed by utilizing SEM. SEM is a robust multivariate technique that explores complex and simultaneous interactions among multiple factors [96].

Based on the estimated SEM results and if needed, data from the literature a calibrated and validated SD model could be formulated allowing us to estimate the likelihood of ACLI in a population [97,98]. In this way, different scenarios could be assessed, and various interventions could be applied to evaluate their impact on ACLI. By means of this approach, effective policies for preventing ACLI could be proposed as a supporting tool. Developing a tool in the form of a simulation model that could provide information on the dynamic interactions among risk factors for ACLI could serve as an interactive model for the prevention of ACLI. Such a model, which predicts the long-term effects of decisions concerning ACLI, can aid coaches, health providers, policymakers, and stakeholders in gaining insight into the problem of ACLI and designing effective strategic plans for its prevention. The incorporation of SD simulation modeling in the understanding of ACLI prevention would offer an opportunity to test plausible interventions.

## 5. Conclusions

The proposed ACLI-CLD constitutes a rigorous conceptual presentation of the dynamic interactions between the intrinsic and extrinsic factors that contribute to ACL injury. It may visually be observed through the proposed ACLI-CLD model that institutional, psychological, neurocognitive, neuromuscular, malalignment factors and trauma history affect neuromuscular control, which may alter the biomechanics of landing, predisposing the ACL to injury. The proposed model also suggests that the risk can increase further if specific environmental and anatomical factors affect the shear forces imposed on the ACL synthesizing a complex framework for the ACL injury etiology.

It is noteworthy that the proposed model is based on state-of-the-art knowledge and agreement among experts and stakeholders on the factors that contribute to ACL injuries. In this way, the ACLI-CLD can serve as a useful tool for the investigation of several “what-if” scenarios and facilitate discussion and interprofessional team decision-making on multiple aspects of the ACL injury system. Further, the proposed ACLI-CLD model serves as the basis for future quantitative research studies incorporating the system dynamics methodology for simulating the propensity for ACL injuries and evaluating plausible prevention strategies prior to implementation.

## Figures and Tables

**Table 1:** GMB process for the formulation of ACLI-CLD.

Steps	Task’s Purpose	Time	Script from Scriptapedia	Actions	Participants
A	To review the primary risk factors for anterior cruciate ligament injury (ACLI) through systematic reviews and pertinent literature	-	-	Create a list of ACLI risk factors for further discussion in the group modeling building workshops	Two members of the modeling Team
B	Group Modeling Building Workshops				
B1	To discuss list of risk factors for ACLI	120′	Variable elicitation	Participants discuss the variables and remove or add variables.	Modeling Team and two members of the stakeholders’ team
B1.1	To develop a CLD for ACLI (ACLI-CLD) using literature as a reference and incorporating the insights gained from the previous GMB session’s discussion	-	-	Developing an initial understanding of the ACLI risk factors’ interplay	One member of the modeling team
B2	To engage in conversation and evaluate the ACLI-CLD with the modeling team	120′	1. Causal Mapping with Seed Structure, 2. Model Review 3. Next steps and closing	Review the ACLI-CLD and propose corrections	Modeling Team
B3	To review ACLI-CLD with the modeling team	60′	1. Model Review, 2. Next Steps and Closing	Review the ACLI-CLD after the corrections and prepare the following steps	Modeling team
B4	To present ACLI-CLD to stakeholders and integrate their perspectives	120′	1. Modeling project community presentation, 2. Model Review	Presentation of the ACLI-CLD to stakeholders, accompanied by a discussion of the ACLI-CLD and its constituent variables	Modeling team and all members of the Stakeholders’ team
B5	To review the ACLI-CLD and incorporate stakeholders’ points of view	90′	Initiating and elaborating a “Causal Loop Diagram” or “Stock and Flow” Model, 2. Model Review	Summarizing the inputs from stakeholders	Modeling Team

Abbreviations: ACLI—anterior cruciate ligament injury, CLD—causal loop diagram, GMB—group modeling building.

## Data Availability

There are no additional data. All the data are presented in this article.
